# Rest energy expenditure is decreased during the acute as compared to the recovery phase of sepsis in newborns

**DOI:** 10.1186/1743-7075-7-63

**Published:** 2010-07-23

**Authors:** Rubens Feferbaum, Cláudio Leone, Arnaldo AF Siqueira, Vitor E Valenti, Paulo R Gallo, Alberto OA Reis, Ary C Lopes, Viviane G Nascimento, Adriana G de Oliveira, Tatiana Dias de Carvalho, Rubens Wajnsztejn, Claudia de Castro Selestrin, Luiz Carlos de Abreu

**Affiliations:** 1 Departamento de Pediatria, Universidade de São Paulo (USP), São Paulo, SP, Brasil; 2 Departamento de Saúde Materno-infantil, Universidade de São Paulo (USP), São Paulo, SP, Brasil; 3 Departamento de Medicina, Disciplina de Cardiologia, Universidade Federal de São Paulo (UNIFESP), São Paulo, SP, Brasil; 4 Departamento de Morfologia e Fisiologia, Faculdade de Medicina do ABC, Santo André, SP, Brasil

## Abstract

**Background:**

Little is known with respect to the metabolic response and the requirements of infected newborns. Moreover, the nutritional needs and particularly the energy metabolism of newborns with sepsis are controversial matter. In this investigation we aimed to evaluate the rest energy expenditure (REE) of newborns with bacterial sepsis during the acute and the recovery phases.

**Methods:**

We studied nineteen neonates (27.3 ± 17.2 days old) with bacterial sepsis during the acute phase and recovery of their illness. REE was determined by indirect calorimetry and VO_2 _and VCO_2 _measured by gas chromatography.

**Results:**

REE significantly increased from 49.4 ± 13.1 kcal/kg/day during the acute to 68.3 ± 10.9 kcal/kg/day during recovery phase of sepsis (P < 0.01). Similarly, VO_2 _(7.4 ± 1.9 *vs *10 ± 1.5 ml/kg/min) and VCO_2 _(5.1 ± 1.7 *vs *7.4 ± 1.5 ml/kg/min) were also increased during the course of the disease (P < 0.01).

**Conclusion:**

REE was increased during recovery compared to the sepsis phase. REE of septic newborns should be calculated on individualized basis, bearing in mind their metabolic capabilities.

## Introduction

As compared with birth the body weight doubles at five months of age and is three-fold greater at the end of the first year of life. Their length increases approximately 25-30 cm in the first year of life, which represents 75 to 85% of the length at birth. As a result of such a rapid growth the requirements of nutrients and energy are higher during the neonatal period (especially preterm) as compared to later in life [1]. The most frequent infections during the neonatal period not only contribute to enhance morbidity and mortality rates [2], but may also impact on the energy and nutrient requirements.

In the presence of severe infection, the metabolic response is characteristically elevated, and in adults energy metabolism is higher following septicemia. Significant changes in protein metabolism and severe proteolysis occurs in muscle and visceral protein. As a consequence of these changes protein re-synthesis of immune factors, such as antibodies and acute phase reactants which the C-reactive protein (CRP) are generated [3,4].

Resting energy expenditure (REE) is the amount of energy expended while at rest in a neutrally temperate environment in the post-absorptive state. The release of energy in this state is sufficient only for vital organs. REE decreases with age and is directly proportional to lean body mass [5-8].

Albeit previous studies assessed the metabolism of adults with sepsis, little is known regarding the metabolic response in septic infants. In addition, conflicting data are available about the nutritional requirements and the energy metabolism of infants [9,10]. Therefore, we aimed to evaluate REE in newborns with bacterial sepsis during the acute and the recovery phases of illness. We hypothesized that the newborn's metabolic demands are increased during recovery, when compared to the acute phase.

## Materials and methods

### Study Population

This study protocol was approved by the University of Sao Paulo Research Ethics Board and subjects were only enrolled following parental consent.

We studied 19 children admitted in the University of Sao Paulo Neonatal Intensive Care Unit with the diagnosis of a bacterial sepsis. The subjects' were 20 ± 17 days old, birth weight of 2966 ± 502 grams and a male:female ratio of 10:9. Except for one (gestational age 35 weeks), all infants were full term.

### Inclusion Criteria

The following inclusion was utilized: 1) age up to 90 days old; 2) birth weight higher than 1500 g; 3) clinical evidence of bacterial sepsis defined according to positive blood culture and/or modified clinical laboratory criteria of Bone [11]. We considered the measurement of C-reactive protein (CRP) higher than 5 mg/liter as suggestive of infection [12]. Other laboratory tests such as cerebrospinal fluid changes suggesting bacterial meningitis [13], chest X-rays with the image of bronchopneumonia were also considered auxiliary criteria for the diagnosis of bacterial sepsis.

### Exclusion Criteria

Infants were excluded if presenting with liver or respiratory failure. The latter was defined or mechanical ventilation.

### Variables analysis

The infant's medical management was determined by their attending physicians. The infants were evaluated time-in two periods: sepsis and recovery phases. The sepsis phase was characterized by a positive blood culture. The recovery phase was characterized by the normalization of blood count, CRP and a negative blood culture. Laboratory exams were performed according to the following techniques:

- Blood culture: collected in media for aerobic and anaerobic bacteria and plated on agar-blood;

- Blood cell count: automated method for the laser hematology counter CELL-DYN 3000^® ^that performs global and differential counts of platelets and formed elements with the confirmation of results for optical microscopy;

- CRP: measured by nephelometric technique using reagents "High sensitive CRP" and "Reagent additional Oumu-15" brand in Behring^® ^device Behring^® ^Nephelometer 100;

- Cerebrospinal fluid: according to the technique proposed by Spina-França, 1971.

Weight was measured daily throughout the hospitalization in an electronic scale accurate to 5 grams, with a capacity of 10 kg, calibrated before each measurement. We calculated Z scores based on weight and chronological age. In order to calculate it we used the ANTHRO 1.01 program of the Nutrition Division of the Center for Disease Control (CDC-USA) and Nutrition Unit/World Health Organization, December, 1990.

Newborns received only parenteral nutrition, which was based on glucose as an energy source, especially in preterm infants, a fact also noted previously [14]. Test measurements were evaluated at least 12 hours after feeding.

The gestational age was calculated by the last menstrual period reported by the mother (Naegele's rule) and when possible we used the somatic Capurro method [15]. The classification of newborns with respect to birth weight and gestational age was based on the Brazilian Ramos curve [16].

Determination of indirect calorimetry was performed in two stages of the child evolution, always by the same investigator:

- On the acute phase of bacterial sepsis after hemodynamic stabilization which corresponds to the flow restoration level;

- On recovery phase of bacterial sepsis, when the newborn was considered clinically and biochemically free of infection by the attending physician, with the registry of the weight gain and at least 10 days after the first testing.

Assessment of indirect calorimetry is part of the routine tests performed in our center for newborns with metabolic and/or severe nutritional disorders requiring nutritional support. Details of our method to measure REE in newborns have been previously published by Cardoso et al [17]. They used the same technique of indirect calorimetry measurement in our study and demonstrated that the REE in malnourished infants was approximately 58.3+/-10.9 kcal/kg on hospitalization. Validation studies have shown the technique to give results equivalent to direct measurements [16,18]. The calorimeter used was developed by Hamamoto [19] and built from the incubator model C-86 of Fanem. Routine calibration was performed prior to each study using a 0.5 L syringe and primary standard calibration gases. Physical activity was assessed by the scale of Scopes and Ahmed [20], considering the scores 0 (sleep, eyes closed) and 1 (eyes open, physically still).

The volume of air in the system was measured geometrically (140 liters), and the air flow around the fan was 75 liters per minute. On the anterior portion of the calorimeter there was a system of three-way taps to collect gas which is made with glass syringe fitted with locks and sealed with silicone. The test measurement involved placing the newborn in the incubator after feeding, keeping the ventilation ports opened, while temperature and humidity of the system and the newborn's home stabilize.

The gas collected (20 mL) were sent to the laboratory and analyzed by GC-35 chromatograph calibrated, the reading of the concentrations of oxygen and carbon dioxide were made in integrating CG processor 300 coupled to the gas chromatograph. One gas sample was needed to get a stable result. VO_2 _and VCO_2 _were corrected to standard conditions of pressure and temperature and we calculated REE using the modified Weir equation [21]:

VO_2 _= Oxygen uptake (ml/Kg/min)

VCO_2 _= Carbon dioxide production (ml/Kg/min)

REE = Resting Energy Expenditure (Kcal/Kg/day)

### Statistical Analysis

Data distribution was evaluated by D'Agostino Pearson normality test. Considering that all variables presented parametric distribution we used paired Student t-test to compare the acute and recovery phase values. The significance level were adopted for 1% (p < 0.01). The statistical analysis was conducted with Sigma Stat software.

## Results

Four infants died during the study period due to worsening of their clinical condition. Table 1 lists the complete blood count, CRP and cultures results for the acute phase, while Table 2 shows similar data relative to the recovery phase.

**Table 1:** Complete blood count, CRP and cultures on acute phase of sepsis.

Subject	Hb (g/dl)	Hct (%)	NI	**Leukocyte/mm**^**3**^	**Platelet/mm**^**3**^	CRP (mg/l)	Identified bacteria
1	8.8	24	0.23	23500	32000	-	HMC: *Staphylococcus *coagulase
2	10.6	30	0.02	12000	31400	9.82	HMC: *Klebsiella pneumoniae*
3	14.4	41	0.20	8000	480000	-	HMC: *Escherichia coli*
4	9.6	27	0.11	18700	72000	36.40	HMC: *Streptococcus pyogenes*
5	13.6	39	0.02	16400	16000	9.30	HMC: *Enterobacter cloacae*
6	13.6	38	0.20	21000	193000	-	HMC: *Streptococcus pyogenes*
7	14.7	42	0.01	14900	560000	-	HMC: *Staphylococcus aureus*
8	18.3	52	0.10	6500	111000	14.20	HMC: *Enterobacter aglomerans*
9	7.8	21.1	0.22	4900	72000	203.00	HMC: *Staphylococcus aureus*
10	12.0	34	0.21	10100	206000	3.30	HMC: *Enterobacter aglomerans*
11	8.5	24	0.02	10300	610000	-	HMC: *Enterobacter cloacae*
12	9.2	26	0.20	11000	72000	132.20	HMC: *Staphylococcus aureus*
13	10.6	32	0.21	20600	144000	57.50	Pus articulation *Staphylococcus aureus*
14	7.9	24	0.04	3700	200000	37.30	LCR: *Neisseria meningitidis*
15	12.5	36	0.20	11800	272000	21.00	HMC: *Corynebacterium sp*
16	10.0	28	0.30	4800	80000	-	HMC: *Enterococcus faecalis*
17	11.5	31	0.30	3900	186000	-	Ascitic liquid: *Pseudomonas aeruginosa*
18	11.1	32	0.20	21700	350000	-	HMC: *Staphylococcus aureus*
19	9.4	27	0.20	24900	429000	-	HMC: *Enterococcus faecalis*

CRP: C-reactive protein; Hb: Hemoglobin; Hct: Hematocrit; NI: Neutrophil Index.

**Table 2:** Complete blood count, CRP and cultures on recovery phase of sepsis.

Case	Hb (g/dl)	Hct (%)	**Leukocyte/mm**^**3**^	NI	**Platelet/mm**^**3**^	CRP (mg/l)	Cultures
1	14.6	42	7900	0.06	200000	2.98	Negative
2	11.2	32	10700	0.04	75000	2.00	Negative
3	12.0	35	10000	0.05	130000	-	-
4	9.3	28	11700	0.06	400000	3.30	Negative
5	13.3	36	11400	0.09	120000	-	Negative
6	12.0	34	12000	0.08	146000	-	-
7	13.1	40	9600	0.05	210000	-	Negative
8	11.5	35	11800	0.01	210000	7.00	Negative
9	11.7	35	12800	0.05	390000	9.00	Negative
10	9.9	28	8500	0.10	250000	3.40	Negative
11	12.5	36	9700	0.07	395000	6.00	Negative
12	10.4	30	8100	0.05	570000	3.40	Negative
13	9.1	27	7400	0.04	319000	8.00	Negative
14	10.6	31	13600	0.10	190000	-	Negative
15	12.2	36	12100	0.10	177000	-	Negative
16	14.6	42	7900	0.06	200000	2.98	Negative
17	11.2	32	10700	0.04	75000	2.00	Negative
18	12.0	35	10000	0.05	130000	-	-
19	9.3	28	11700	0.06	400000	3.30	Negative

CRP: C-reactive protein; Hb: Hemoglobin; Hct: Hematocrit; NI: Neutrophil Index.

Table 3 presents the clinical profile of all subjects.

**Table 3:** Clinical profile of each newborn during acute phase of sepsis.

*Case*	*Infection*	*Hypothermy* *(T<36°C)*	*Hyperthermy* *(T>37,5°C)*	*Tachycardia* *(>120 bpm)*	*Tachypnea (>40/min)*	*Antibiotics*
***1***	YES	YES	NO	YES	YES	Vancomycin and Cefotaxime
***2***	YES	NO	NO	YES	YES	Ceftazidime and Imipenem
***3***	YES	NO	YES	YES	NO	Ceftriaxone
***4***	YES	YES	NO	YES	YES	Penicillin and Amicacine
***5***	YES	NO	YES	YES	YES	Vancomycin and Cefotaxime
***6***	YES	YES	NO	YES	YES	Ampicillin and Amicacine
***7***	YES	NO	YES	YES	YES	Oxacillin
***8***	YES	YES	NO	NO	YES	Vancomycin and Cefotaxime
***9***	YES	NO	YES	YES	YES	Vancomycin and Ceftriaxone
***10***	YES	NO	NO	YES	YES	Penicilyin and Amicacine
***11***	YES	NO	NO	YES	YES	Vancomycin and Cefotaxime
***12***	YES	NO	YES	YES	YES	Vancomycin and Ceftriaxona
***13***	YES	NO	NO	YES	YES	Vancomycin and Ceftriaxona
***14***	YES	NO	YES	YES	YES	Penicillin and Amicacine
***15***	YES	NO	YES	YES	YES	Vancomycin and Cefotaxime
***16***	YES	YES	NO	YES	YES	Oxacillin and Amicacine
***17***	YES	YES	NO	YES	YES	Vancomycin and Imipenem
***18***	YES	YES	NO	YES	YES	Vancomycin and Cefoxitin
***19***	YES	NO	YES	YES	YES	Meropenen

We noted no changes in newborns oxygen saturation and axillary temperature. The respiratory quotient (RQ) data showed an average of 0.7 during sepsis, which is indicative of the predominant combustion of fat and ketone bodies, resulting in lower CO_2 _production.

**Figure 1:**
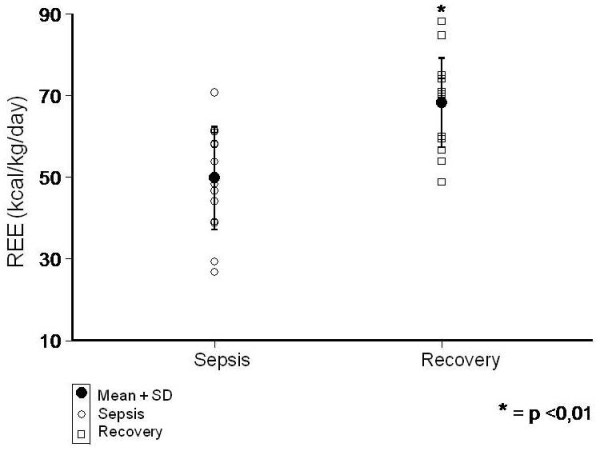
**Comparison of REE (kcal/kg/day) between sepsis and recovery phase**.

**Figure 2:**
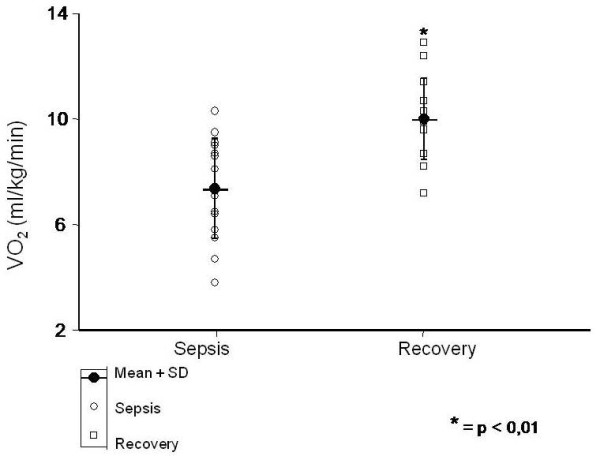
**Comparison of VO_2 _(ml/kg/min) between sepsis and recovery phase**.

**Figure 3:**
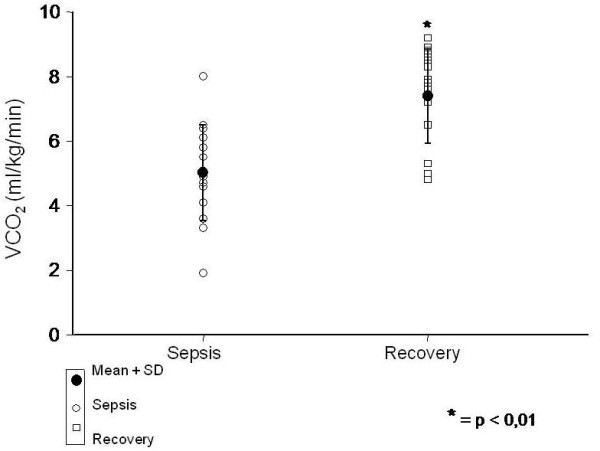
**Comparison of VCO_2 _(ml/kg/min) between sepsis and recovery phase**.

During the acute phase of sepsis the REE was 49.4 ± 13.1 kcal/kg/day and on recovery it significantly increased to 68.3 ± 10.9 kcal/kg/day (p < 0.01) (Figure 1). Moreover, VO_2 _(7.4 ± 1.9 *vs *10 ± 1.5 ml/kg/min) (Figure 2) and VCO_2 _(5.1 ± 1.7 *vs *7.4 ± 1.5 ml/kg/min) (Figure 3) also increased during the recovery phase (p < 0.01). Those findings support the hypothesis that the whole body metabolic rate is enhanced after sepsis recovery.

Table 4 shows data relative to age and weight changes during sepsis and recovery phase.

**Table 4:** Age (days) and weight (g) at sepsis and recovery phase of sepsis.

Case	Age (days)			Weight (g)			
	Hospitalization	Sepsis	Recovery	Birth	Hospitalization	Sepsis	Recovery
1	24	31	41	3030	3295	3570	3620
2	6	28	39	3070	2820	2535	2750
3	13	14	25	3230	3580	3480	3630
4	18	30	50	2630	2730	2685	3520
5	1	10	24	1800	1680	1695	2000
6	1	2	13	2750	2760	2580	2600
7	22	25	35	2730	3640	3565	3750
8	9	11	26	3500	3000	3080	3040
9	16	20	42	3000	3130	2780	2890
10	31	33	44	3640	3580	3570	3980
11	51	57	69	3300	4500	4710	4965
12	15	21	44	3440	3660	4130	3180
13	27	33	54	2580	2760	2490	2990
14	63	69	81	1960	4015	3880	4365
15	4	7	28	3530	3300	3700	3570
16	30	34	-	3200	3900	3885	-
17	6	11	-	2930	2725	3070	-
18	1	45	-	3470	3470	2690	-
19	35	38	-	3150	2430	2510	-

**Mean**	19.6	27.3	41	2996.8	3209.2	3189.7	3390
**Median**	16	28	41	3070	3295	3080	3520
**SD**	17.1	17.2	17.8	502.3	647.9	730.9	737.3
**Maximum**	63	69	81	3640	4500	4710	4965
**Minimum**	1	2	13	1800	1680	1695	2000

## Discussion

We endeavored to evaluate the REE in newborn with bacterial sepsis at acute and recovery phases. We reported that at the recovery phase REE was increased compared to the acute sepsis phase, probably due to the resumption of growth. Albeit we investigated a small number of newborns, statistical analysis showed significant differences between acute and recovery phases of sepsis.

In adults with bacterial sepsis it has been shown that caloric and protein needs increased by as much as 150% of normal basal metabolism rate [22]. In this age group equations for calculating caloric requirements are often used; one of the best known is the Harris-Benedict. However, this equation tends to overestimate the caloric needs [23].

We reported that VO_2 _and VCO_2 _were increased at the recovery phase of sepsis. There are few studies where the energy requirements in children and especially in newborns with bacterial sepsis were evaluated and the clinically utilized data in neonates was extrapolated from adult values [24]. The application of various predictive equations of REE for children under mechanical ventilation as the Harris-Benedict and Talbot equations formulas overestimate the caloric requirement [25,26].

Nevertheless, other studies have reported decreased basal metabolic rate in sick children. Powis et al [27] evaluated children undergoing major surgery and did not report an increase in basal metabolic rate during the postoperative period of six newborns with necrotizing enterocolitis. Chwals [28] evaluated the overfeeding (over nutrition) associated to the metabolic effects which occur when nutritionally supporting extremely ill children. The author concluded that the caloric requirement of these children are unpredictable and modified by disease, age, previous nutritional status and type of nutrients (especially parenteral use) with varied specific dynamic action. Therefore, the caloric requirements of critically ill children should be assessed individually and measured by appropriate techniques.

In our study we measured REE through indirect calorimetry. Several techniques are used to evaluate the basal metabolism rate or REE. The most used is the indirect calorimetry, due to its accuracy, portability, comfort and safety for the patient, even in seriously ill subjects [29]. The equipment and technique utilized in the present study were developed by Hamamoto [19] and are especially appropriate for newborns and young children. The method validation was performed by measuring REE in newborns and comparing the results with data from studies that used the same technique. During our study there were no changes in the oxygen saturation and axillary temperature of the newborns. The technique is utilized in our center as part of the patient assessment and it is used frequently for children with metabolic disorders and/or to determine adequacy of nutritional support [19].

We found that REE averaged 49.4 ± 13.1. These values are very close to those of a previous study (51.7 ± 6.21 kcal/kg/day), which used the same apparatus and technique. In the present study, the highest REE during sepsis was 70.7 kcal/kg/day. Considering that in normal children the average REE is 58 kcal/kg/day [25], only six study subjects showed REE values above the normal range. Even in one case the REE was 70.7 kcal/kg/day, which represents only 20% above the highest reference value.

We reported that some of the newborns died during our study. The analysis of REE data in these subjects showed that two neonates exhibited REE values (51.7 ± 6.21 kcal/kg/day) within the adopted reference range. However, we observed that newborns showing significantly lower REE values were more likely to have a poor outcome. In fact, the infant who presented one of the lowest REE (27.8 kcal/kg/day) quickly evolved to demise. Briassoulis et al [25] proposed that the measurement of basal metabolism rate may be a prognostic factor for mortality: the lower the value, the greater the odds of a poor prognosis.

Based on our results, we found no hypermetabolism, differently from what is established in adults with bacterial sepsis where REE is estimated at 100-150% higher than the values of basal metabolism. The same can be said about the VO_2 _and VCO_2_. The average VO_2 _of our study was 7.4 ± 1.9 ml/kg/min, similar to the value of 7.41 ± 0.88 ml/kg/min verified by Hamamoto [19]. The VCO_2 _during acute phase of sepsis averaged 5.1 ± 1.7 ml/kg/min, which was below the average of 6.36 ± 1.04 reported by the authors. The lower values likely relate to changes in energy metabolism and burning fat and preferential ketone bodies, which frequently occurs in malnourished children and low-calorie-protein intake. This assumption is also supported by the analysis of the RQ. The RQ = 0.7 we found during sepsis is indicative of the predominant combustion of fat and ketone bodies, resulting in lower CO_2 _production.

The proposed explanation for our results regarding REE, i.e. the absence of hypermetabolism energy has not been adequately discussed in the literature. Nonetheless, some studies related to bacterial sepsis in children are relevant to our analysis. Uzel and Neyzi [30] studied thyroid function in severely infected children and they reported that the values of thyroxine (T_4_) and thyroid-stimulating hormone (TSH) were comparable to normal controls, whereas triiodothyronine (T_3_) were much lower and triiodothyronine (reverse rT_3_) were higher. Mendoza-Morfin et al [31] investigated infants with bacterial sepsis and observed reduction of T_4 _and T_3 _associated to increase of rT_3 _and TSH. After the recovery of sepsis these values returned to normal in children who survived. The authors suggested that through these hormonal changes positively contributed to prevent sepsis-induced catabolism. Furthermore, Hatherill et al [32] evaluated adrenal function in infants with septic shock and demonstrated adrenal insufficiency in 52% of cases. The consequence of this fact was reflected in higher mortality, severe hypotension and more frequent use of vasoactive drugs to maintain blood pressure. Togari et al [33], on the same line of research, demonstrated a decrease of plasma cortisol in children with endotoxic shock. The analysis of these studies showed a reduction of T_3_, cortisol and catecholamines mainly caused by adrenal insufficiency, an important fact because the triiodothyronine and catecholamines are hormones that promote the body thermogenesis. Probably the interpretation of our results with respect to the absence of hypermetabolism energy in children during the acute phase of sepsis is explained by hormonal changes described above.

The present study has the following caveats. Firstly, we decided not to use a control group since energy expenditure in healthy newborns is well described in the literature [34] and in this investigation we opted to comparatively evaluate the changes in the same infant over time (acute sepsis phase and recovery phase). Secondly, parenteral [28], enteral and oral feeding influence REE [35] and we evaluated newborns which received parenteral feeding. Thirdly, we did not perform any multivariate analysis in order to assess the study variables although this could have provided more detailed information regarding REE. We suggest further studies to evaluate this issue. Fourthly, newborns did not require higher FIO_2 _and were not mechanically ventilated. These results are not supported by Kreymann et al [36], who found a normal or only slightly elevated REE in adult patients with severe sepsis or septic shock but a hypermetabolic response in patients with uncomplicated sepsis. It is possible that this apparent conflict can be explained by standardization approach described in our method section.

In summary, during the acute phase of bacterial sepsis REE was significantly reduced, as compared with the subsequent recovery phase of sepsis. We recommend that REE during the acute phase of sepsis be individualized for each child, taking into account the severity of the disease, the degree of malnutrition and the type of energy substrate used with respect to the metabolic capacity of the child.

## Competing interests

The authors declare that they have no competing interests.

## Authors' contributions

RF, VEV, LCA, CL, AAFS, AGO and TDC designed research, analyzed data, and wrote the paper. RF, VEV, PRG, AOAR, RW and LCA carried out the statistical analysis and participated in design the manuscript. All authors read and approved the final manuscript.
